# A Rare Case of Cotard’s Delusion Treated With Oral and Long‐Term Injectable Antipsychotics: A Case Report

**DOI:** 10.1155/crps/5079901

**Published:** 2026-07-27

**Authors:** Shivani Senthil Kumar, Hayley Silverstein, Amoolya Vayalapalli, Taylor Pigg, Oliver M. Glass, Hardeep Singh

**Affiliations:** ^1^ Department of Graduate Medical Education Research, Northeast Georgia Medical Center, Gainesville, Georgia, USA, nghs.com; ^2^ Department of Psychiatry, Northeast Georgia Medical Center, Gainesville, Georgia, USA, nghs.com; ^3^ Medical College of Georgia, Augusta University, Augusta, Georgia, USA, augusta.edu

**Keywords:** Cotard’s delusion, Cotard’s syndrome, long-acting injectable antipsychotic, nihilistic delusion syndrome, paliperidone palmitate, schizophrenia

## Abstract

Cotard’s delusion is a relatively infrequent neuropsychiatric condition that presents as a form of extreme and abnormal thinking where an individual believes that they are dead, do not exist, or have lost parts of their body or internal organs. The primary descriptions of Cotard’s delusion were associated with severe depressive illness; however, Cotard’s delusion may also present in individuals with schizophrenia. Because Cotard’s delusion often appears as part of an array of psychotic symptoms, it can be misdiagnosed or underdiagnosed in hospitals. As such, many reported cases of Cotard’s delusion have been treated with electroconvulsive therapy (ECT), after failing to respond to pharmacologic treatments. However, there are some reported cases of Cotard’s delusion that have achieved remission using medication.

We describe the case of a 40‐year‐old male with a several‐year history of schizophrenia who developed an acute psychotic episode characterized by nihilistic delusions consistent with Cotard‐like symptoms. The individual believed that he was dead, that he did not exist, and that his throat had been cut. During this episode, the individual expressed somatic delusional thoughts in addition to the previously mentioned nihilistic delusional thoughts. Upon the onset of his psychotic episode, the individual demonstrated a unique euphoric mood, which seemed to be related to his beliefs regarding being dead. The patient was treated with oral risperidone, followed by long‐acting injectable (LAI) paliperidone palmitate, resulting in the gradual resolution of symptoms during a short hospitalization.

This case highlights the limited number of documented cases of Cotard’s delusion in patients with schizophrenia and the potential role of LAI antipsychotic agents as a maintenance strategy for patients with schizophrenia and significant compliance issues who develop Cotard delusions.

## 1. Introduction

Cotard’s delusion, first recognized by Jules Cotard in 1880 and later named after him, is characterized by symptoms including delusions of negation, feelings of nonexistence, severe depressive features, and detachment from reality [1, 2]. Some people with Cotard’s delusion will believe that they are deceased. Others will be convinced that certain parts of the body have stopped working or that some or all their organs have stopped functioning [3]. Eventually, many will go so far as to deny their own existence. Those who deny their own existence can be expected to abandon basic self‐care activities (eating/drinking/hygiene), which include taking care of themselves. Although Cotard’s delusion is most associated with a severe depressive illness, there are reports of individuals with schizophrenia and other psychoses who report nihilistic delusions.

About 1% of the global population is affected with schizophrenia, but some cases are rare and very significant from a clinical standpoint [4]. One such case is Cotard’s delusion, which consists of false beliefs about death. Systematic studies of Cotard’s delusion cases suggest that among 1321 inpatient consultations, 0.62% of psychotic patients had Cotard’s delusion [5]. Those who have the delusion believe that they are dead or that their internal organs are nonexistent. Even though Cotard’s delusion is most often reported in severe mood disorders, it has also been noticed in schizophrenia, particularly when individuals are experiencing episodes of acute psychosis [6]. In these instances, the delusion may show up without the usual depressive effect and instead accompany bizarre delusions, flat or euphoric moods, and impaired insight [7]. Its occurrence with schizophrenia is thought to be rare, especially when depression is not also a factor, which makes these cases rather distinct and important to document. More widely, Cotard’s delusion is linked to many conditions, including major depression with psychotic features, bipolar disorder, and schizoaffective disorder. It is also associated with a range of serious neurological conditions like epilepsy or traumatic brain injury [8]. Despite being a serious condition, Cotard’s delusion is not recognized as a distinct diagnosis in DSM‐5 [9]. The treatments for this condition usually involve antipsychotic medication [9]. Electroconvulsive therapy (ECT) is reserved for people with Cotard’s delusion who are resistant to other treatments [10]. In addition to the affective presentation, several case studies have documented Cotard’s delusion in schizophrenia, in both individuals who exhibit no clear signs of depression and in whom various reactions were observed toward the use of typical and atypical antipsychotics and ECT, as documented, for example, in Morgado et al. [7], Shiraishi et al. [11], Huarcaya‐Victoria et al. [12], Bott et al. [13], and Caliyurt et al. [14]. In addition, numerous review articles and algorithms that summarize data from approximately 300 or so previously published cases of Cotard’s delusion have concluded that this phenomenon is present in mood disorders, schizophrenia, and several other neurologic diseases and is likely responsive to combinations of antidepressant medications, antipsychotic medications, and ECT. In a more recent study, Fusick et al. [9] emphasize the use of two psychotropic medications to avoid ECT in some patients who exhibit severe manifestations of Cotard’s delusion.

While not formally recognized in the DSM‐5 as a standalone diagnosis, Cotard’s delusion is detailed in an ever‐growing base of clinical literature as a condition that is effectively treatable [9]. Reports of treatment efficacy predominantly feature antipsychotic medications, most commonly those from the atypical class. Risperidone [15], olanzapine [9], fluoxetine [9], and aripiprazole [16] have all been reported to have favorable results. In cases resistant to treatment or when delusions pose a danger to life, ECT has been employed widely and with remarkable results [10]. One published case report involves a patient with schizophrenia and Cotard’s delusion who was found to have a positive reaction to a 2‐week course of aripiprazole [16]. Delusional intensity was greatly reduced, and emotional responsiveness was markedly improved [16]. This demonstrates that even without ECT, oral antipsychotics alone can lead to some select cases of rapid symptom resolution.

This case report shows a full recovery from Cotard’s delusion using paliperidone palmitate—a long‐acting injectable (LAI) antipsychotic after initial stabilization using oral risperidone. Prior published cases have documented the use of oral antipsychotics and ECT in the treatment of Cotard’s delusion; however, no published case has specifically described the transition to an LAI as a compliance‐driven maintenance strategy for a nonadherent patient with schizophrenia who develops Cotard’s delusion. Therefore, this case demonstrates that LAIs may be an effective and practical maintenance option in patients with schizophrenia who are not adherent to medication, where relapse and rehospitalization are primarily due to the lack of adherence to medication.

## 2. Case Presentation

A man in his 40s was admitted to the emergency department complaining of a migraine and joint pain. The patient’s past medical history was significant for high blood pressure and schizophrenia. The patient also had a history of using multiple substances, including marijuana, cocaine, alcohol, and methamphetamine. However, the patient’s urine drug screen upon admission tested negative for drugs. At the beginning of the initial psychiatric interview, the patient stated that he had experienced several disturbing thoughts. The patient believed that he had died and that his throat had been cut. The patient had previously expressed these thoughts during two episodes of severe depression, one of which occurred while he was hospitalized. The patient had previously been prescribed mirtazapine, risperidone, and sertraline, but had discontinued this regimen prior to his admission. Additionally, there was no documented family history of psychiatric disease. The patient described several delusional thoughts. He expressed nihilistic beliefs in that he believed that he was dead. He also stated that he had somatic (related to the body) and persecutory (fear of being harmed or attacked) beliefs, including that his throat had been cut or that his body had been harmed. Although he denied hearing voices or seeing things, he appeared to be responding to internal stimuli. His emotional responses remained limited, and his thought patterns remained somewhat disordered. Overall, this clinical presentation is consistent with an acute exacerbation of schizophrenia with delusional content.

A psychomotor exam was conducted, which demonstrated normal psychomotor activity, good eye contact, and a cooperative attitude. His speech was normal in rhythm, latency, and tone, with a linear and goal‐directed thought process, while his mood was depressed and his affect was constricted. Though the patient denied suicidal ideations (SI), homicidal ideations (HI), auditory hallucinations (AH), visual hallucinations (VH), and delusions, he appeared to be responding to internal stimuli and exhibited disorganized behavior; paranoia and delusions were noted as well. He denied any symptoms of mania, such as elevated moods or euphoria. The mental status examination revealed that the patient was alert and oriented to person, place, time, and situation (A/Ox4). His short‐ and long‐term memory was intact, without gross deficits. Attention span, concentration, language, abstraction, and fund of knowledge were all grossly intact. No significant cognitive deficits were observed. Insight and judgment were assessed as fair.

Given the presence of delusions regarding death and prior psychiatric history with schizophrenia, the patient required inpatient psychiatric stabilization. The treatment plan included initiating risperidone 1 mg p.o. at bedtime, monitoring psychosis, supportive psychotherapy, and obtaining collateral information from family and friends. Given the patient’s chronic tobacco use, the plan was to limit this habit with a nicotine patch, tobacco counseling, and to monitor cravings. The hospitalist was consulted for medical management, and lisinopril was initiated for hypertension.

During the second day in the psychiatric unit, it was noted that the patient had recently relocated from Texas after his mother moved for work, and therefore, he had not yet established psychiatric care. He later reported that his mother is deceased. The patient remained disorganized throughout the interview, frequently blocking his thoughts and believing that he was deceased. He denied SI, HI, AH, and VH.

On re‐evaluation, he displayed normal motor activity, appropriate eye contact, and cautious behavior. His speech was soft and slow in rhythm and rate. His thought process remained linear and goal‐directed; his mood was anxious, and his judgment was impaired. Despite denying hallucinations, he was still reacting to his own ideas and demonstrated persistent delusions. Due to ongoing psychosis and the patient’s poor insight, his risperidone dose was increased to 2 mg orally at bedtime. Psychotherapy was used to assist the patient with anxiety and to support a positive relationship, while facts from the patient’s family and friends were gathered to understand the patient and their needs moving forward.

During his third day in the hospital, the patient still demonstrated symptoms of schizophrenia with Cotard’s delusion and paranoia. He complained that his brother had broken his leg in several places and that an injury to his throat was causing him to lose his voice. He repeated the belief that he was not alive. An evaluation of his current symptoms was carried out during another psychiatric examination. The patient said he does not feel secure where he is, as he previously was poisoned and does not know anyone at the hospital. Even so, he stated that his medications were off and did not know the purpose of the medications nor did he suggest how they would improve his situation. His lack of trust in the staff appeared to cause increased anxiety and limited his cooperation with treatment. Despite denying AH and VH, Cotard’s delusion was still present, and paranoia continued to be a prominent symptom for the patient. He showed limited emotional involvement, with poor insight and impaired judgment. As symptoms continued despite antipsychotic treatment, risperidone was increased to twice a day, starting with 2 mg, to control psychotic symptoms. The patient remained hospitalized for close monitoring, and the team continued to look for collateral information from the family or prior providers.

Day four brought little progress in paranoia, and the patient seemed more cheerful, yet his disorganization did not change. The delusion had decreased, and now the patient felt discomfort from blurry eyes and felt that his skin was torn. He appeared very messy, had difficulty making eye contact, and spoke more slowly. While thoughts were not organized, they wandered less, and the patient had no AH/VH or delusions during the interview. Judgment and insight remained impaired, but his mood improved, and he appeared better overall. Given the observed symptom improvement at the current dose (2 mg of risperidone twice daily), the dose was not reduced. The team decided that the patient should try paliperidone palmitate when the blood pressure was within a healthy range.

On day five, the patient continued to show improvement in mood, Cotard’s delusion, and paranoia. He claimed that he slept well and had fewer negative thoughts, yet issues with his vision and skin remained. He told us that he often questioned if he was dead and wanted to learn about cars and medicine. He appeared clean, wore the right clothes, was friendlier socially, and maintained appropriate eye contact. His speech was quiet, and he responded to situations after some time had passed. He denied the ideas of AH/VH and SI/HI, and there were no signs of delusions or paranoia. Once stability was achieved, the team administered the second paliperidone palmitate injection (234 mg) and reduced the risperidone dose to 1 mg twice daily.

**Figure 1:**
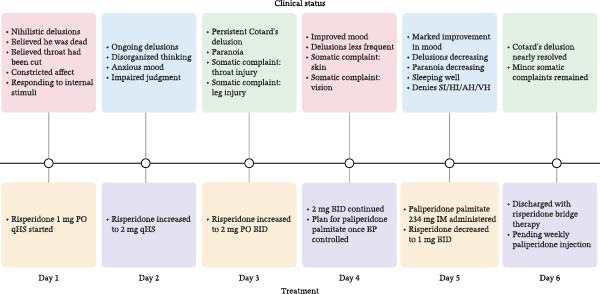
Timeline of the patient’s clinical course during hospitalization. Clinical status (top) and therapeutic interventions (bottom) during the 6‐day inpatient hospitalization, demonstrating the progressive resolution of Cotard’s delusion following risperidone titration and transition to long‐acting injectable paliperidone palmitate.

By day 6, the patient was discharged with a 2 mg (twice‐daily) risperidone prescription, which was meant to be used only until his scheduled 1‐week paliperidone palmitate injection. When the patient was discharged, his Cotard’s delusion had nearly resolved, and the remaining symptoms were minor somatic complaints. The patient’s hospital course and therapeutic interventions are summarized in Figure 1.

## 3. Discussion

The rare neuropsychiatric condition known as Cotard’s delusion is marked by beliefs that are of a nihilistic nature—that one is dead, has lost internal organs, or no longer exists [17]. Most frequently, it has been seen in patients who have a severe and unremitting form of depression or psychotic disorders such as schizophrenia [13]. Some researchers believe that when this condition occurs in the context of schizophrenia, it may be due to the right‐frontal lobe [13]. Although Cotard’s delusion is not formally recognized in DSM‐5, academic literature recognizes its pattern of symptom progression [9]. While typically linked to serious mental health conditions (depression), with extreme symptoms, there are some instances where people with schizophrenia may believe that they do not exist, have died, or have missing internal body parts. Such beliefs often arise during an active episode of psychosis and/or alongside other delusions (such as a belief that someone is out to get them or a specific part of their body is “bad”). Thus, while such beliefs may be described as “Cotard‐like” when they arise in people who suffer from schizophrenia, they should not be seen as evidence of an independent diagnosis, but instead as manifestations of the many forms of disorganized thinking which characterize a full‐blown psychotic break.

The usual procedure in the diagnostic evaluation is to carry out a comprehensive psychiatric assessment together with a urine drug screen to eliminate the possibility of substance‐induced psychosis [18]. Acute psychotic episodes that may mimic symptoms like Cotard’s Delusion have been linked with substances such as methamphetamine, cocaine, lysergic acid diethylamide, psilocybin mushrooms, synthetic cannabinoid receptor agonists, and amphetamine [18].

The initial presentation of the patient, a male in his 40s with schizophrenia, involved somatic delusions (e.g., throat injury and damaged limbs) that developed into the final conviction that he was already dead. This presentation aligned with previous reports of Cotard symptoms evolving over a few days to weeks during an acute psychotic episode. Standard clinical recommendations were followed, which included a comprehensive psychiatric interview; a urine drug screen was done to rule out substance‐induced psychosis, especially since there was a reported history of substance use.

Cotard’s delusion treatment methods rely heavily on case studies and series; thus, atypical antipsychotics are commonly prescribed and have demonstrated positive effects regarding symptom reduction across all forms of mood and psychosis‐related symptoms (including Cotard’s delusion) within schizophrenia, such as those treated with sulpiride, risperidone, and aripiprazole, with or without ECT. Most patients will require a combination of medications, and when the above treatments do not provide adequate symptom relief, some may benefit from ECT. Most of what is known about treatments for Cotard’s delusion comes from either single‐case studies or small case series. Antipsychotics are commonly used, especially in patients who experience Cotard’s delusion in conjunction with a psychotic disorder like schizophrenia. There are documented instances of ECT resulting in substantial improvement in patients with severe or unresponsive Cotard’s delusion. In this case, the patient exhibited improvement after switching to long‐acting paliperidone palmitate injections. In a recent article by Fusick et al. [9], they recommend using dual medication approaches (e.g., olanzapine and fluoxetine) to avoid the use of ECT in severe Cotard’s presentations with nihilistic delusions after titrating oral risperidone. The patient described in their report remained stabilized thereafter [9].

Our patient’s response to treatment began with a gradual titration of oral risperidone to 2 mg twice daily, resulting in a clear decrease in both the severity and frequency of Cotard’s delusion symptoms. This stabilization was then maintained with the LAI paliperidone palmitate. The patient’s discharge was completed after the near resolution of nihilistic beliefs concerning death and the belief that his throat had been cut. Only minor somatic concerns were present upon discharge. As reported previously in other cases of Cotard’s delusion, it is possible for some patients to achieve resolution of their delusional beliefs through pharmacological treatment alone. This case is noteworthy for documenting the use of paliperidone LAI as a maintenance strategy in a chronically ill, previously nonadherent patient with schizophrenia who developed Cotard’s delusion, a combination not described in the literature to date.

We also wish to emphasize that, while this case report is limited to data from an inpatient setting and does not include formal outpatient follow‐up data, we are unable to confirm whether the remission of Cotard’s delusion was sustained following discharge based on the information currently available. Accordingly, future case reports and prospective studies should include structured follow‐up intervals to better characterize the durability of LAI‐based maintenance strategies in this population.

The LAI antipsychotic is generally recommended for the patient with schizophrenia with a history of poor compliance (nonadherence) or moderate compliance with oral medications. There has been a substantial amount of observational and cohort study data that demonstrate that adherence in patients on LAIs is better than that in patients on oral antipsychotics and is also associated with a lower rate of relapse, hospitalization, and treatment discontinuation than that with oral antipsychotics [19]. LAI antipsychotics are effective for treating schizophrenia and other related psychotic disorders because they improve outcomes by leading to better adherence or compliance with medication use, relapse stabilization, and disease progression stabilization [20]. Moreover, extended interval formulations, such as paliperidone palmitate (3 or 6 months), have been demonstrated to influence relapse rates compared to other formulations of antipsychotic medications in the literature, as well as maintaining remission, in this case, for patients with schizophrenia, and possibly assisting when converting patients from serial shorter‐acting formulations [21]. Conversely, discontinuation or suboptimal adherence to LAIs severely compromises outcomes. Mirror‐image and cohort studies indicate that interrupting LAI therapy can increase the relapse risk more than tenfold, leading to frequent hospitalizations and prolonged recovery phases [22]. Each relapse episode not only delays symptomatic recovery but also contributes to neurobiological deterioration and social dysfunction [22]. LAIs foster better treatment adherence by markedly reducing the daily burden of oral dosing with longer dosing intervals, such as monthly, quarterly, or biannual injections [23]. These forms of medication also support continual interaction with the patient and ongoing relationships to help identify and address emerging psychiatric symptoms as they arise [23]. Additionally, LAIs have been associated with improved patient satisfaction, functional outcomes, and quality of life [24].

In this case, the injectables provided the patient with stable symptom control during the inpatient hospitalization; however, no formal follow‐up data are available to confirm sustained symptom control after discharge. Most of the medical literature is either about the use of ECT or oral monotherapy. However, this case report describes the use of a LAI antipsychotic in a patient with Cotard’s delusion. In this case, treatment with LAI paliperidone palmitate was associated with clinical improvement and may represent a potential maintenance strategy for selected patients with Cotard’s delusion and schizophrenia who meet criteria for LAI therapy. A limitation of this case is that the patient exhibited heterogeneous delusional content (both somatic and persecutory) as well as typical nihilistic delusions of Cotard’s delusion.

This case demonstrates the importance of confirming a diagnosis through the exclusion of secondary causes, especially when self‐neglect is so severe that it could lead to life‐threatening health problems. Physical examinations played key roles in verifying the diagnosis. Next, this case highlights the utility of antipsychotic medications, particularly LAIs (paliperidone), when trying to achieve optimal pharmacologic results with a noncompliant patient. ECT also remains an important tool for treating treatment resistance [7]. This case highlights the symptoms and challenges of recognizing Cotard’s delusion as a form of psychosis that is quite severe and occurs within the schizophrenia spectrum [6]. It is emphasized that care considerations require that boundaries between schizophrenia and mood disorders should be maintained, but also recognize them within this spectrum [6]. Together, this case provides an addition to the body of literature on Cotard’s delusion in schizophrenia, as it demonstrates that a series of oral risperidone therapy followed by long‐acting paliperidone palmitate (LAI) therapy may induce and maintain significant reductions in Cotard’s delusional beliefs in a chronically psychotic and historically noncompliant patient, which is complementary to previously documented oral‐only and two‐medication treatments.

## 4. Conclusion

Cotard’s nihilistic delusions were identified in a patient experiencing a chronic exacerbation of schizophrenia. The patient was treated effectively using pharmacotherapy with risperidone orally and then LAI paliperidone palmitate as the primary maintenance therapy. Antipsychotic therapy alone resulted in a significant resolution of the patient’s nihilistic beliefs; no ECT was required. This case emphasizes the importance of identifying nihilistic delusions in patients with schizophrenia.

Having timely recognition and intervention by psychiatry was essential not just for improvement but also in preventing a potentially life‐threatening condition. This patient’s treatment with a LAI antipsychotic was key because it addressed his nonadherence to a prescribed medication regimen in a way that allowed him to sustain important clinical gains following his discharge. Finally, this case illustrates the need for physicians to monitor key indicators of the treatment response, such as speech and self‐care.

## Funding

The authors have nothing to report.

## Ethics Statement

This manuscript describes a single‐patient case report and therefore did not require Institutional Review Board (IRB) review or approval.

## Consent

The patient provided written informed consent for their clinical information to be used in this case report and was informed that any identifying information would be anonymized. The patient understands that this case report may be published and will be used for educational and research purposes. Patient‐signed consent form can be submitted upon request from reviewer/editor after patient health information is redacted.

## Conflicts of Interest

The authors declare no conflicts of interest.

## Data Availability

The data that support the findings of this study are available from the corresponding author upon reasonable request.
